# Cardiovascular Benefits With Favorable Renal, Amputation and Hypoglycemic Outcomes of SGLT-2 Inhibitors in Type 2 Diabetes From the Asian Perspective: A Population-Based Cohort Study and Systematic Review

**DOI:** 10.3389/fendo.2022.836365

**Published:** 2022-03-07

**Authors:** Chun-Ting Yang, Zi-Yang Peng, Yi-Chi Chen, Huang-Tz Ou, Shihchen Kuo

**Affiliations:** ^1^ Institute of Clinical Pharmacy and Pharmaceutical Sciences, College of Medicine, National Cheng Kung University, Tainan, Taiwan; ^2^ Department of Pharmacy, College of Medicine, National Cheng Kung University, Tainan, Taiwan; ^3^ Division of Metabolism, Endocrinology & Diabetes, Department of Internal Medicine, University of Michigan Medical School, Ann Arbor, MI, United States

**Keywords:** DPP-4 inhibitor, SGLT-2 inhibitor, Asia, cardiovascular, renal, amputation, all-cause death, hypoglycemia

## Abstract

**Objective:**

We assessed the effects of sodium glucose cotransporter-2 inhibitors (SGLT2is) versus dipeptidyl peptidase-4 inhibitors (DPP4is) in a large real-world Asian cohort with type 2 diabetes (T2D) and performed a systematic review with integrating the present study findings to provide up-to-date evidence from the Asian perspective.

**Methods:**

New users of SGLT2is or DPP4is were identified from the Taiwan’s National Health Insurance Research Database and followed until 2018. Primary outcomes were hospitalization for heart failure (HHF) and three-point major adverse cardiovascular event (3P-MACE; namely, myocardial infarction [MI], stroke, or cardiovascular death). Other outcomes included all-cause death, chronic kidney disease (CKD), amputation, and hospitalized hypoglycemia. Subdistribution hazard models were employed to assess treatment-associated clinical outcomes.

**Results:**

A total of 21,329 SGLT2i and DPP4i propensity-score-matched pairs were analyzed. SGLT2is versus DPP4is showed lower risks of HHF (hazard ratio [95% CI]: 0.52 [0.45–0.59]), 3P-MACE (0.62 [0.55–0.70]), MI (0.63 [0.50–0.79]), stroke (0.60 [0.51–0.70]), all-cause death (0.57 [0.49–0.67]), CKD (0.46 [0.43–0.50]), amputation (0.64 [0.42–0.98]), and hospitalized hypoglycemia (0.54 [0.45–0.64]). Our results were consistent with findings from a systematic review.

**Conclusion:**

Among Asian patients with T2D, SGLT2is versus DPP4is showed benefits for several clinical outcomes. More research is warranted to explore the heterogeneous treatment effects of SGLT2is and DPP4is by race/ethnicity.

## Introduction

According to the Standards of Medical Care in Diabetes by the American Diabetes Association, the use of sodium glucose cotransporter-2 inhibitors (SGLT2is) is recommended after first-line metformin treatment failure for patients with type 2 diabetes (T2D) and comorbid atherosclerotic cardiovascular diseases (CVDs), heart failure (HF), or chronic kidney disease (CKD) (1). Substantial evidence on cardiorenal benefits associated with SGLT2is has promoted its use in routine care (2, 3). As a result, a growing number of studies have focused on the head-to-head comparative effectiveness and safety associated with the use of SGLT2is versus other glucose-lowering agents (GLAs), where dipeptidyl peptidase-4 inhibitors (DPP4is) are commonly used as a comparator drug for SGLT2is. DPP4is are the most prescribed oral GLAs for T2D patients who failed metformin therapy in clinical settings (3, 4) owing to their relatively neutral effects on the risks of hypoglycemia, weight gain, and CVDs (1).

Network meta-analyses (5, 6) and real-world studies (7–11) have demonstrated that SGLT2is versus DPP4is lower the risks of hospitalization for HF (HHF), major adverse cardiovascular event (MACE), and all-cause death. However, Asian populations are underrepresented in these studies. Asian populations with T2D have several differences compared with Caucasian populations such as younger onset of T2D, higher prevalence of stroke and CKD, and lifestyle dissimilarities (e.g., high rice consumption) (12, 13). Hence, the generalizability of results from studies that primarily comprised Western populations to Asian settings is limited. Moreover, most previous Asian studies of T2D patients mainly focused on cardiovascular or mortality outcomes associated with the use of SGLT2is without analyzing safety outcomes of treatment (e.g., amputation, hospitalized hypoglycemia) (14–20). Additionally, adjustment for competing risk of death to clinical outcomes of interest (e.g., CVDs) was not considered in previous analyses, leading to biased estimates of relative hazards of study outcomes (14–17, 21, 22).

The present study evaluated the real-world comparative effectiveness of SGLT2i versus DPP4i treatment on a comprehensive spectrum of clinically important outcomes, namely, CVDs, all-cause death, CKD, amputation, and hospitalized hypoglycemia in a large Asian cohort with T2D. A systematic review was further performed and integrated with our study findings to provide up-to-date evidence on the real-world outcomes of SGLT2i versus DPP4i use in Asian populations with T2D.

## Materials and Methods

### Data Source

The National Health Insurance Research Database (NHIRD) was utilized for this population-based, retrospective cohort study. The NHIRD provides de-identified and individual-level longitudinal claims data of outpatient visits, inpatient admissions, emergency department visits, and prescription information for each beneficiary enrolled in the National Health Insurance (NHI) program, which covers over 99% of the Taiwanese population (23). This study was approved by the Institutional Review Board of National Cheng Kung University Hospital (A-EX-109-035).

### Cohort Identification

The incident new-user, active-comparator design was employed in this study (24). That is, T2D patients who newly initiated study drugs (SGLT2is or DPP4is) (i.e., incident new-users) in 2017 were first identified from the NHIRD. This design was applied to mitigate time-related bias such as prevalent-user bias and survivor bias. Based on an active-comparator design, the effect of SGLT2is as the study drug of interest was compared to that of DPP4is as an active drug used in clinical practice, instead of ‘no treatment’ (non-users). Such a design is commonly used in observational studies to increase the comparability between study groups and mitigate the effects of confounding by indication/contraindication to ensure the internal validity of study findings (24).

Moreover, to avoid short-term SGLT2i or DPP4i use, we included only stable users of the study drugs, defined as those with (i) at least three sequential refills of SGLT2is or DPP4is after treatment initiation and (ii) a prescription gap between any two consecutive refills of fewer than 30 days. The first date of SGLT2i or DPP4i use in 2017 was defined as the index date. Second, the stable users who have been exposed to either SGLT2is or DPP4is in the year prior to the index date were excluded to ensure the inclusion of incident new users of the study drugs in the study cohort. Also, those with exposure to both SGLT2is and DPP4is at the index date were excluded. Third, we excluded those with chronic renal dialysis or renal transplantation in the year before the index date to avoid the inclusion of patients with severe renal impairments.

Finally, to enhance the between-group comparability of the study groups, the propensity score (PS) matching technique based on baseline patient characteristics was applied. The PS for each patient was estimated using a logistic regression model where drug exposure (SGLT2is versus DPP4is) was treated as the dependent variable, and a series of patient characteristics, including demographics at the index date, diabetes-related complications in the year prior to the index date, and previous exposure to GLAs and CVD-related medications in the year prior to the index date, were measured as the independent variables. Additionally, to minimize the potential heterogeneity in baseline patient renal function, several surrogate indicators from previous studies (25) and recommendations of clinical experts were measured and included in the estimation of PS. These indicators were the status of participation in a pay-for-performance program for pre-end-stage renal disease (ESRD) at the index date, which was a proxy for patients with an estimated glomerular filtration rate (eGFR) level of less than 45 ml/min/1.73 m^2^, and exposure to metformin, acarbose, or sulfonylureas within 90 days prior to the index date, which were proxies for patients with eGFR levels of less than 30, 25, and 15 ml/min/1.73 m^2^, respectively. SGLT2i and DPP4i users were 1:1 matched using the 5-to-1 digit greedy PS matching approach (26). A flowchart of the study cohort selection is shown in **Supplementary Figure 1**. The operational definitions of variables considered in the PS estimation and the kernel density curves of PS distributions for the two study groups are detailed in **Supplementary Table 1** and **Supplementary Figure 2**, respectively.

### Operational Definitions of Drug Exposure and Study Outcomes

Exposure to SGLT2is or DPP4is was measured using the World Health Organization Anatomical Therapeutic Chemical Classification system. Primary outcomes were HHF and 3P-MACE (namely, non-fatal myocardial infarction [MI], non-fatal stroke, or cardiovascular death). Secondary outcomes included 4P-MACE (comprising 3P-MACE and HHF), non-fatal MI, non-fatal stroke, all-cause death, CKD, and safety outcomes of treatment, namely, amputation and hospitalized hypoglycemia. All study outcomes were identified using the International Classification of Diseases, Ninth Revision, Clinical Modification (ICD-9-CM) and the International Classification of Diseases, Tenth Revision, Clinical Modification (ICD-10-CM) diagnosis or procedure codes (**Supplementary Table 2**). The validity of using these codes to identify study outcomes in the NHIRD has been reported elsewhere (23). The mortality status was confirmed using the Cause of Death File of the NHIRD. Each patient was followed from the index date until the occurrence of the study outcomes of interest, death, lost to follow-up from the NHI program, or December 31, 2018, whichever came first (i.e., intention-to-treat [ITT] scenario), in the primary analyses.

### Statistical Analyses

Differences in baseline characteristics between the study drug groups before and after PS matching were tested using the standardized mean difference (SMD), where an absolute value of 0.10 or greater indicates a significant between-group difference (27). Considering the competing risk of death to study outcomes, associations of using SGLT2is versus DPP4is with study outcomes were estimated using subdistribution hazard models and presented as subdistribution hazard ratios (SDHRs) with 95% CIs (28). Sensitivity analyses based on the as-treated (AST) scenario were also performed, where patients were followed from the index date until discontinuation, switching to or adding on of the other study drug, occurrence of study outcomes, death, lost to follow-up in the NHI program, or December 31, 2018, whichever came first.

Subgroup analyses were performed to evaluate whether the treatment effects of study drugs differed by baseline patient characteristics, namely, age, gender, diabetes duration, histories of CVDs, HF, and CKD, and previous exposure to insulin, which have been considered in the subgroup analyses in previous studies (14–16, 19, 22). To ensure the comparability of patient characteristics between SGLT2i and DPP4i users within each subgroup stratum, the PS matching and subdistribution hazard model analyses were redone within each stratum. A two-tailed *p*-value of less than 0.05 was considered to indicate a statistically significant difference. All analyses were conducted using SAS software version 9.4.

### Systematic Review

A systematic review of SGLT2is versus DPP4is on clinical outcomes in Asian populations with T2D was performed. Two authors (CTY and YCC) independently searched for studies on PubMed and Embase from the inception of the databases to May 13, 2021 using the framework of PICO. It included 1) population (P): patients with type 2 diabetes, 2) intervention (I): SGLT2is, 3) comparison (C): DPP4is, and 4) outcome (O): clinical outcomes (e.g., CVDs). After this search, the two authors (CTY and ZYP) independently reviewed the title and abstract of each identified article to determine the eligibility of study for further full-text review. The detailed search strategies and keywords are shown in **Supplementary Table 3**. A flowchart of the study selection that follows the PRISMA flow diagram (2009 version) (29) is shown in **Supplementary Figure 3**.

## Results

A total of 21,329 PS-matched pairs of SGLT2i and DPP4i users were identified (**Supplementary Figure 1**). The kernel density curves of PS distributions for the two study groups before and after PS matching are shown in **Supplementary Figure 2**. After PS matching, the baseline characteristics between the two study groups were comparable (**Table 1**). In the primary analysis (ITT scenario), the mean follow-up of the study cohort was 1.6 years.

**Table 1:** Baseline characteristics of study population stratified by study drugs (SGLT2is and DPP4is) before and after propensity score matching.

Characteristics	Before PSM			After PSM		SMD^†^
	DPP4is	SGLT2is	SMD^†^	DPP4is	SGLT2is	
Number of subjects	50,051	22,925		21,329	21,329	
Age at index date (years, mean ± SD)	64.68 ± 12.58	57.12 ± 11.47	**−0.63**	58.55 ± 11.73	57.91 ± 11.20	0.00
Male (%)	53.42%	57.23%	0.08	57.00%	56.70%	0.00
Number of GLAs subjects were exposed to within one year before index date (mean ± SD)	1.52 ± 0.99	1.63 ± 1.00	**0.10**	1.58 ± 1.08	1.58 ± 1.05	0.01
Duration of diabetes at index date (year, mean ± SD)	8.48 ± 3.20	8.35 ± 3.20	−0.04	8.35 ± 3.20	8.34 ± 3.21	0.00
**Proxies of renal function before index date (%)**						
Participants in pre-ESRD program within one year before index date	1.42%	0.20%	**−0.14**	0.19%	0.22%	0.01
Metformin prescribed within 90 days before index date	48.01%	44.97%	−0.06	46.11%	45.69%	−0.01
Acarbose prescribed within 90 days before index date	10.38%	13.23%	0.09	12.47%	12.63%	0.00
Sulfonylureas prescribed within 90 days before index date	4.26%	5.68%	0.07	5.22%	5.44%	0.01
**Diabetes-related complications within one year before index date (%)**						
Nephropathy	27.31%	20.80%	**−0.15**	21.01%	21.04%	0.00
Neuropathy	9.42%	9.08%	−0.01	8.94%	9.01%	0.00
Retinopathy	8.18%	7.73%	−0.02	7.92%	7.75%	−0.01
Peripheral vascular disease	4.46%	3.36%	−0.06	3.19%	3.46%	0.02
Cerebrovascular disease	8.40%	4.14%	**−0.18**	4.39%	4.37%	0.00
Cardiovascular disease	19.70%	19.28%	−0.01	18.65%	19.00%	0.01
Heart failure	4.89%	3.41%	−0.07	3.42%	3.49%	0.00
Acute myocardial infarction	1.54%	1.81%	0.02	1.77%	1.69%	−0.01
Ischemic heart disease	12.20%	12.71%	0.02	12.29%	12.45%	0.00
Diabetic ketoacidosis	0.40%	0.12%	−0.06	0.17%	0.11%	−0.02
Hypoglycemia	1.73%	0.28%	−**0.15**	0.30%	0.30%	0.00
**GLAs prescribed within one year before index date (%)**						
Metformin	57.12%	53.55%	−0.07	54.56%	54.42%	0.00
Sulfonylureas	46.02%	45.61%	−0.01	46.65%	45.99%	−0.01
Meglitinides	8.26%	5.48%	−**0.11**	5.74%	5.64%	0.00
Thiazolidinediones	12.26%	17.18%	**0.14**	16.21%	16.52%	0.01
Acarbose	14.48%	17.64%	0.09	16.27%	17.01%	0.02
GLP1 RAs	0.28%	2.17%	**0.17**	0.63%	0.68%	0.01
Insulins	13.63%	20.96%	**0.19**	17.50%	18.13%	0.02
**CVD-related medication history within one year before index date (%)**						
Lipid-lowering medications	69.05%	79.59%	**0.24**	78.82%	78.57%	−0.01
Alpha-blockers	5.83%	4.06%	−0.08	4.54%	4.17%	−0.02
Beta-blockers	34.12%	33.53%	−0.01	33.66%	33.42%	−0.01
RAAS agents	61.14%	61.52%	0.01	61.95%	61.67%	−0.02
Diuretics	20.65%	13.09%	−**0.20**	13.83%	13.59%	−0.01
Calcium channel blockers	38.26%	27.43%	−**0.23**	28.94%	28.59%	−0.01
Anti-arrhythmics	3.21%	2.14%	−0.07	2.57%	2.22%	−0.02
Cardiac glycosides	1.70%	1.18%	−0.04	1.19%	1.22%	0.00
Vasodilators	13.57%	12.78%	−0.02	12.61%	12.70%	0.00
Antiplatelets	38.50%	34.66%	−0.08	34.48%	34.88%	0.01
Anticoagulants	2.82%	1.86%	−0.06	1.82%	1.95%	0.01

PSM, propensity score matching; DPP4is, dipeptidyl peptidase-4 inhibitors; SGLT2is, sodium glucose cotransporter-2 inhibitors; SMD, standardized mean difference; SD, standard deviation; GLAs, glucose-lowering agents; ESRD, end-stage renal disease; GLP1 RAs, glucagon-like peptide-1 receptor agonists; CVD, cardiovascular disease; RAAS, renin–angiotensin aldosterone system.

^†^SMD values in bold indicate significant differences in baseline characteristics between the two study groups (absolute value of SMD ≥0.1).

**Table 2** shows the event rates and SDHRs of study outcomes associated with the use of SGLT2is versus DPP4is in the primary analysis. Compared with DPP4is, the use of SGLT2is was associated with a 48% reduced risk for HHF (SDHR: 0.52, 95% CI [0.45, 0.59]) and 38% reduced risk for 3P-MACE (0.62 [0.55, 0.70]). For other cardiovascular and mortality outcomes, the relative hazards on 4P-MACE, non-fatal MI, non-fatal stroke, and all-cause death were 0.58 [0.53, 0.64], 0.63 [0.50, 0.79], 0.60 [0.51, 0.70], and 0.57 [0.49, 0.67], respectively. The use of SGLT2is versus DPP4is was associated with a significantly lower risk of CKD (0.46 [0.43, 0.50]), and safe profiles on amputation (0.64 [0.42, 0.98]) and hospitalized hypoglycemia (0.54 [0.45, 0.64]). The results of AST scenario analyses were consistent with the primary analysis findings (**Supplementary Table 4**).

**Table 2:** Event rates and hazard ratios of clinical outcomes associated with use of SGLT2is versus DPP4is (intention-to-treat analyses).

	SGLT2is (n = 21,329)		DPP4is (n = 21,329)		SDHR of SGLT2is versus DPP4is (95% CI)
	Number of events	Event rate (events/100 pys)	Number of events	Event rate (events/100 pys)	
**Primary outcomes**					
HHF	349	1.06	671	2.05	0.52 (0.45, 0.59)
3P-MACE^†^	409	1.24	656	1.98	0.62 (0.55, 0.70)
**Secondary outcomes**					
4P-MACE^‡^	686	2.09	1,168	3.58	0.58 (0.53, 0.64)
Myocardial infarction	122	0.37	193	0.58	0.63 (0.50, 0.79)
Stroke	263	0.80	437	1.33	0.60 (0.51, 0.70)
All-cause death^§^	248	0.75	433	1.30	0.57 (0.49, 0.67)
Chronic kidney disease	979	3.19	2,003	7.00	0.46 (0.43, 0.50)
Amputation	35	0.11	55	0.17	0.64 (0.42, 0.98)
Hospitalized hypoglycemia	189	0.57	352	1.07	0.54 (0.45, 0.64)

SGLT2is, sodium glucose cotransporter-2 inhibitors; DPP4is, dipeptidyl peptidase-4 inhibitors; pys, person-years; SDHR, subdistribution hazard ratio; HHF, hospitalization for heart failure; MACE, major adverse cardiovascular event.

^†^3P-MACE included non-fatal myocardial infarction, non-fatal stroke, or cardiovascular death.

^‡^4P-MACE included non-fatal HHF, non-fatal myocardial infarction, non-fatal stroke, or cardiovascular death.

^§^Hazard ratio of all-cause death was estimated using the Cox proportional hazard model analysis instead of subdistribution hazard model analysis.

**Figure 1:**
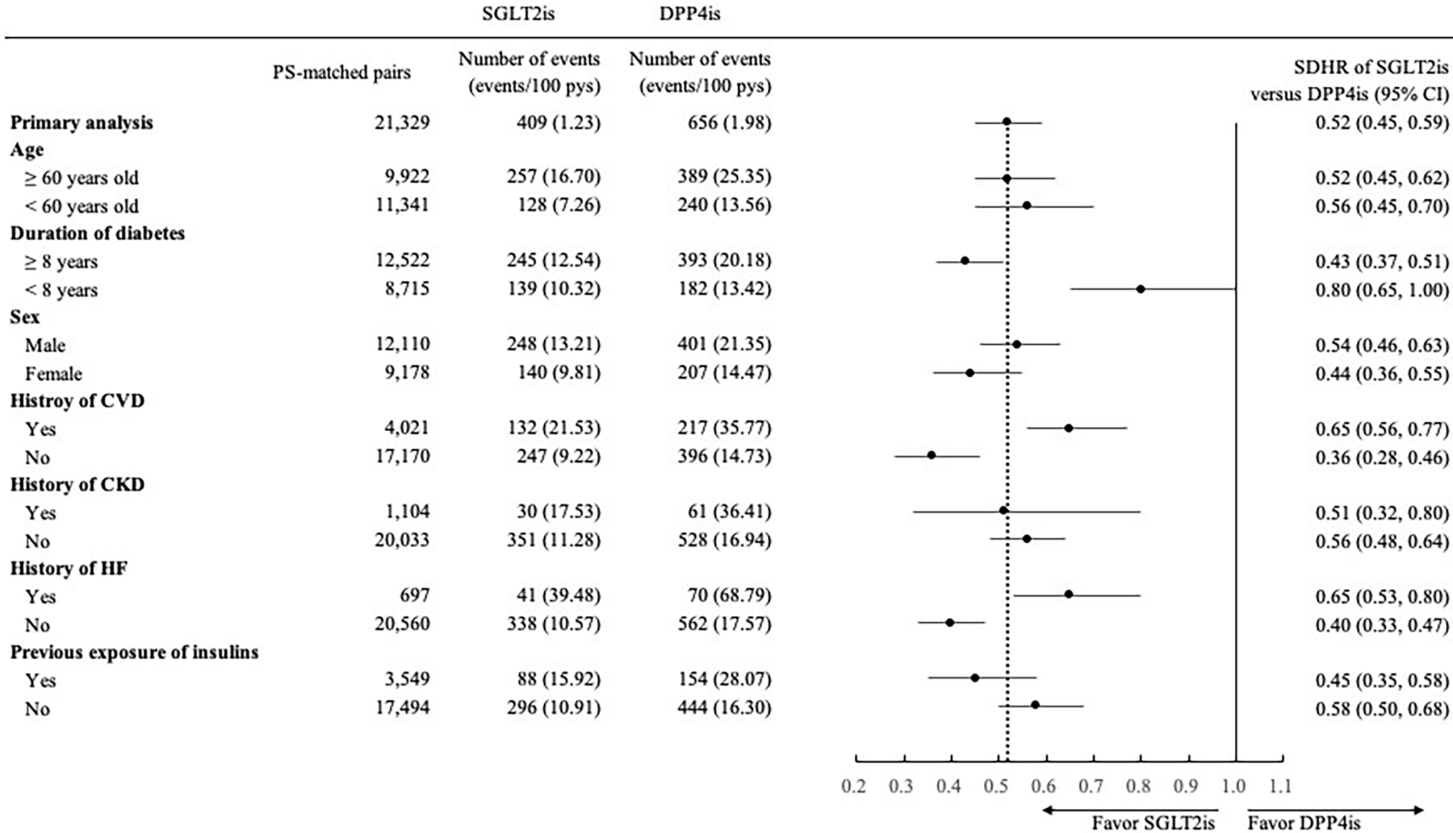
Forest plot of subgroup analyses for hospitalization for heart failure associated with use of SGLT2is versus DPP4is. SGLT2is, sodium glucose cotransporter-2 inhibitors; DPP4is, dipeptidyl peptidase-4 inhibitors; PS, propensity score; pys, person-years; SDHR, subdistribution hazard ratio; CI, confidence interval; CVD, cardiovascular disease; CKD, chronic kidney disease; HF, heart failure.

**Figure 2:**
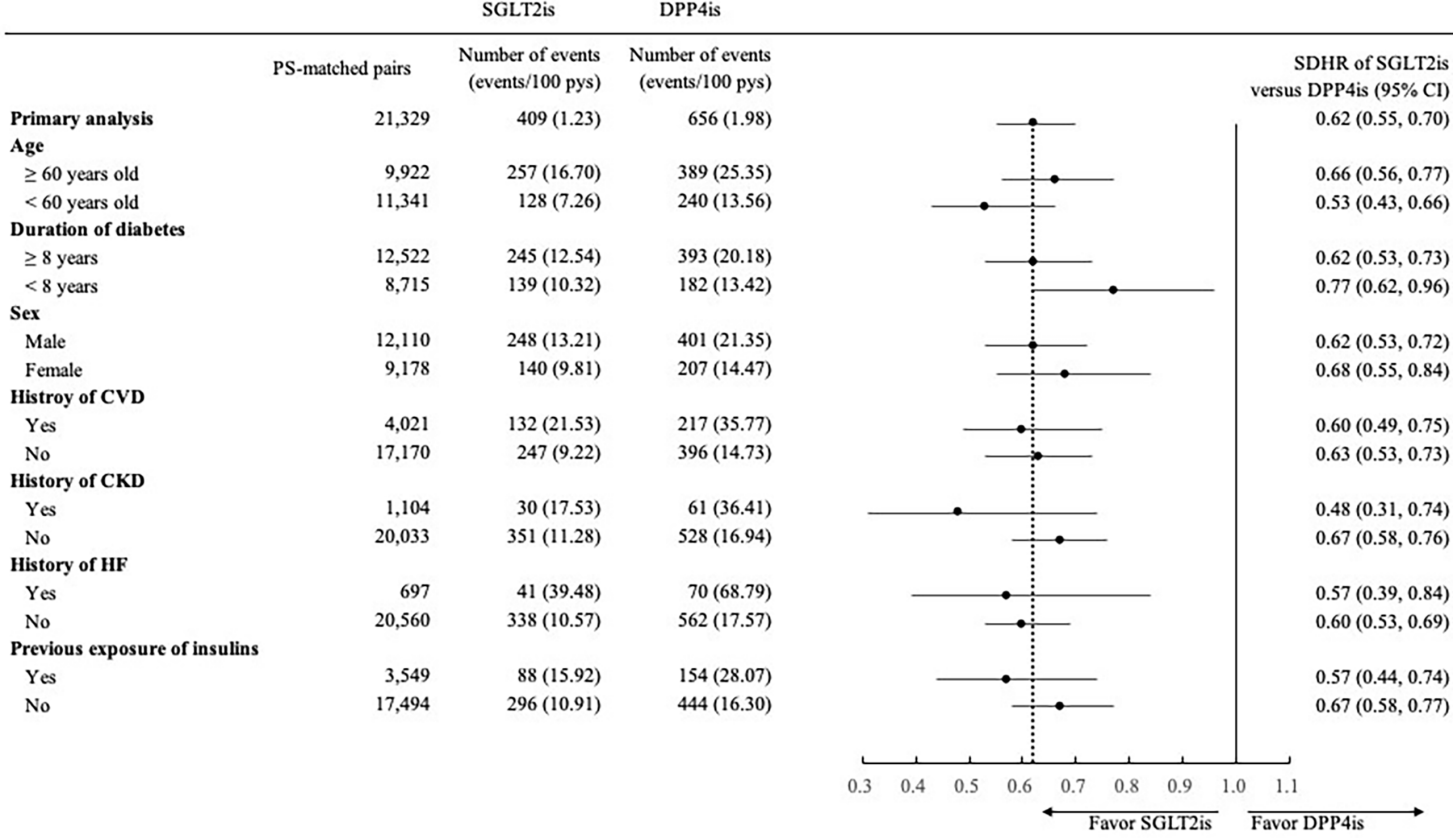
Forest plot of subgroup analyses for 3-point major adverse cardiovascular event (3P-MACE) associated with use of SGLT2is versus DPP4is. SGLT2is, sodium glucose cotransporter-2 inhibitors; DPP4is, dipeptidyl peptidase-4 inhibitors; PS, propensity score; pys, person-years; SDHR, subdistribution hazard ratio; CI, confidence interval; CVD, cardiovascular disease; CKD, chronic kidney disease; HF, heart failure. 3P-MACE included non-fatal myocardial infarction, non-fatal stroke, and cardiovascular death.

The findings of subgroup analyses were generally consistent with those observed in the primary analyses (**Figures 1**, **2**), except for a non-significantly lower risk of HHF when using SGLT2is compared with DPP4is among patients with a diabetes duration of fewer than 8 years (0.80 [0.65, 1.00]).

### Systematic Review

Eleven studies were included in the full-text review, among which three were multinational studies and eight were single-country studies from South Korea, Japan, and Taiwan. All these studies used a retrospective cohort design, utilized population-based databases, and applied PS techniques (e.g., PS matching or weighting) to adjust for imbalanced baseline patient characteristics between treatment groups. In these studies, HHF and all-cause death were the two most common study outcomes. Significant beneficial results were consistently observed across studies, with 14–42% (19, 21) and 15–66% (11, 17) of risk reductions for HHF and all-cause death associated with the use of SGLT2is versus DPP4is, respectively. A 31–77% reduced risk of ESRD or CKD for the use of SGLT2is versus DPP4is was also reported in these studies (14, 15, 17). However, the results for stroke and MI varied across studies. Other outcomes (e.g., hypoglycemia, urinary tract or genital infection, amputation) were also reported, but the number of the studies was relatively limited. The major characteristics and primary findings of each study are summarized in **Supplementary Table 5**.

## Discussion

The present study evaluated the real-world effects of SGLT2is versus DPP4is in a large Asian cohort with T2D and revealed that the use of SGLT2is was associated with significantly reduced risks of HHF, MACE, all-cause death, and CKD compared with DPP4is. Lower risks associated with the use of SGLT2is on the safety outcomes of amputation and hospitalized hypoglycemia, which were not assessed in the previous studies of Asian populations, were also observed. These favorable outcomes with SGLT2i use were consistently observed in subgroups stratified by various patient characteristics, which ensures the robustness of our findings as supportive evidence to the existing literature (11, 14–22, 30) shown in the systematic review to promote the rational use of SGLT2is in a diverse group of real-world Asian patients with T2D.

### Comparison of Reduced Risks of CVDs and All-Cause Death Associated With SGLT2i Use for T2D in This Study and Existing Literature

The reduced risks of CVDs and all-cause death associated with the use of SGLT2is versus DPP4is observed in this study of an Asian T2D cohort are consistent with current evidence for general T2D populations (5–7, 11, 14, 15, 19, 20). Specifically, the reduction of about 48% in HHF risk associated with SGLT2is versus DPP4is found in this study (**Table 2**) falls within the range of estimates reported in previous multinational cohort studies (8–11, 14, 15), which ranged from 18% (14) to 57% (10). Also, the treatment benefit of SGLT2is versus DPP4is on 3P-MACE revealed in this study is comparable to the results of several previous real-world studies (8–11), although the results in the studies of Patorno et al. (7) and Pasternak et al. (based on ITT analysis) (9) did not reach statistical significance. Additionally, the relative hazard of all-cause death when using SGLT2is compared with DPP4is in our analysis (i.e., 0.57 [95% CI: 0.49–0.67]) is close to estimates reported in previous studies (7–11, 14, 15), which ranged from 0.59 (8) to 0.80 (9).

### Potential Benefit on Reduced Risk of Stroke Associated With SGLT2is in Asian Populations With T2D

A non-significantly lower risk of stroke associated with SGLT2is versus DPP4is has been reported in previous analyses of non-Asian populations with T2D (8–10). However, the present study found a significantly reduced stroke risk with the use of SGLT2is in an Asian T2D cohort, which is consistent with the literature on Asian patient populations (11, 15, 17, 21). Specifically, the CVD-REAL 2 study (11), a multinational cohort study conducted in North American, Europe, and Asia, reported that the relative hazard of stroke associated with SGLT2is versus DPP4is in the Korean subpopulation was 0.83 (95% CI: 0.77–0.91) while the significant benefit was not revealed in patients from other countries. Another multinational study that comprised countries from Europe and Asia showed a substantially lower risk of stroke when using SGLT2is versus DPP4is (HR: 0.54 [0.37–0.78]) in the analysis of a Japanese subpopulation but not found in the analysis of the overall study cohort (15).

Although the underlying mechanisms of the potential benefit of SGLT2i treatment for stroke remain unclear, the benefit may be driven by the SGLT2i-associated beneficial effect on reducing the risk of atrial fibrillation (AF) (18, 31). AF is a well-known independent risk factor for stroke; i.e., patients with AF have a 4.4-fold elevated risk of stroke compared with those without AF in the T2D population (32). AF and stroke have been characterized differently between non-Asian and Asian populations in terms of epidemiology and treatment-associated clinical outcomes (33–37). It is thus worthwhile to further assess whether the effect of SGLT2is on the risk of stroke differs across races/ethnicities. This would be crucial for Asian populations, who have a generally higher prevalence of stroke (37), greater stroke-related mortality, and more disability-adjusted life years compared with Western populations (38), to provide compelling real-world evidence to facilitate treatment decisions in Asian settings.

### Other Clinical Outcomes Associated With SGLT2i Use in T2D

The renal benefit (i.e., lower CKD risk) associated with SGLT2is versus DPP4is found in this study is consistent with current evidence (15, 17, 39, 40). A comparable risk of hospitalized hypoglycemia between SGLT2is and DPP4is was also observed in this Asian cohort. Additionally, we are the first to evaluate the amputation outcome of SGLT2i versus DPP4i use among a general T2D population in Asia and found a significantly lower amputation risk of SGLT2i versus DPP4i use. Existing literature on general T2D populations from Western countries have reported a non-significant difference in amputation risk for SGLT2i versus DPP4i use (41–43). However, the external validity of these findings for Asian populations is of concern because the incidence of amputation (44) and the risk factors (e.g., PAD, neuropathy, foot ulcers) associated with the development of amputation (45–47) vary by ethnicity. For example, Young et al. reported that Asian populations with diabetes had the lowest relative risk of amputation compared with other ethnicities (e.g., Caucasian, African American, Hispanic) (45). Although one previous study from Asia reported a significantly lower amputation risk associated with SGLT2i versus DPP4i use, their study patients were restricted to T2D patients with concomitant PAD, which might limit the generalizability of their results to general T2D patients (22). Therefore, the present study extends current knowledge about the effects of SGLT2is on the risk of amputation for general T2D populations in Asia.

### Study Strengths and Limitations

In this real-world study, the reduced risks of HHF and 3P-MACE associated with the use of SGLT2is versus DPP4is were corroborated by a series of sensitivity and subgroup analyses. Favorable results of SGLT2i use across different clinical outcomes were found, supporting the rational use of SGLT2is in Asia. Moreover, our study design adopted several methodological refinements to overcome the limitations commonly seen in previous studies. These refinements included applying subdistribution hazard model analysis to adjust for competing risk of death to study outcomes (e.g., CVDs), implementing surrogate indicators for patient baseline renal function in the estimation of PS to obtain more comparable study drug users, and identifying study patients from 2017 when SGLT2is were commonly prescribed in usual practice in Taiwan to avoid selection bias in our study cohort. With these efforts, this study thus provides more precise and up-to-date estimates for the effects of SGLT2i treatment for Asian populations to promote its rationale use in Asian settings.

Several study limitations should be acknowledged. First, like other observational studies using administrative claims data, unmeasurable confounding effects attributable to the lack of clinical laboratory (e.g., HbA1c) and behavioral (e.g., smoking status) data might exist although great efforts had been made to minimize these effects. For example, several variables that may be associated with the unmeasurable confounders and reflected the baseline patient disease status and severity (e.g., the presence of diabetes-related complications and previous pattern of GLA use) were carefully measured and adjusted in the PS matching. Still, the caution should be made while interpreting the study results due to potential residual confounding effects. Second, the PS matching enhanced the comparability between the study groups but may also limit the generalizability of our study findings to the patients whose characteristics are similar with those of PS-matched DPP4i and SGLT2i pairs. However, we re-iterated the analyses in different subgroups with various clinical characteristics and found that the results of primary and subgroup analyses were consistent. Moreover, the patient characteristics of the present study and those of previous studies that assessed treatment effects of SGLT2is versus DPP4is in Asian populations (11, 14, 15) were generally comparable. Therefore, our study findings might be applicable to the Asian populations with T2D. Third, the length of study follow-up period may not be enough for measuring chronic clinical outcomes. Specifically, the end-stage renal outcomes (i.e., ESRD, renal dialysis) of SGLT2is versus DPP4is were not further analyzed due to limited number of these events, despite a SGLT2i-associated benefit on the risk of CKD revealed in the main analysis. Also, the event sizes of individual CVD outcomes, including myocardial infarction and stroke, were too limited to perform subgroup analyses. Fourth, the number of patients with amputation events was limited, so further analyses on amputation outcomes stratified by patient clinical conditions were not permitted; this is an area for future research. Lastly, the generalizability of our study findings might be limited to Asian countries with universal healthcare coverage.

## Conclusions

Among a real-world Asian cohort with T2D, the use of SGLT2is versus DPP4is was associated with significantly lower risks of HHF, MACE, all-cause death, and CKD, and safe profiles on amputation and hospitalized hypoglycemia. Future research is warranted to explore heterogeneous treatment effects of SGLT2is on clinical outcomes (e.g., stroke, amputation) stratified by patient characteristics (e.g., races/ethnicities) to corroborate our study findings and offer evidence for personalized medicine.

## Data Availability Statement

The data analyzed in this study is subject to the following licenses/restrictions: Data sharing is not applicable to this study as data management and analysis were only allowed to be conducted in Health and Welfare Data Science Center in Taiwan for data privacy and safety concerns. Requests to access these datasets should be directed to huangtz@mail.ncku.edu.tw.

## Ethics Statement

The studies involving human participants were reviewed and approved by the Institutional Review Board of National Cheng Kung University Hospital. Written informed consent for participation was not required for this study in accordance with the national legislation and the institutional requirements.

## Author Contributions

CTY designed the study, performed the review, analyzed and interpreted the data, and wrote the manuscript. ZYP designed the study, performed the review, analyzed and interpreted the data, and wrote the manuscript. YCC performed the review and reviewed the manuscript. HTO provided study materials, designed the study, interpreted the data, and wrote the manuscript. SK designed the study, interpreted the data, and reviewed/edited the manuscript. All authors listed have made a substantial, direct, and intellectual contribution to the work and approved it for publication.

## Funding

This project was supported by grants from the Ministry of Science and Technology in Taiwan (grant MOST 109-2320-B-006 -047-MY3) (HTO). The funder had no role in the design and conduct of the study; collection, management, analysis, and interpretation of the data; preparation, review or approval of the manuscript; and decision to submit the manuscript for publication.

## Conflict of Interest

The authors declare that the research was conducted in the absence of any commercial or financial relationships that could be construed as a potential conflict of interest.

## Publisher’s Note

All claims expressed in this article are solely those of the authors and do not necessarily represent those of their affiliated organizations, or those of the publisher, the editors and the reviewers. Any product that may be evaluated in this article, or claim that may be made by its manufacturer, is not guaranteed or endorsed by the publisher.

## References

[1] American Diabetes Association. 9. Pharmacologic Approaches to Glycemic Treatment: Standards of Medical Care in Diabetes—2021. Diabetes Care (2021) 44(Suppl.1):S111–24. doi: 10.2337/dc21-S009 33298420

[2] Eberly LA Yang L Eneanya ND Essien U Julien H Nathan AS. Association of Race/Ethnicity, Gender, and Socioeconomic Status With Sodium-Glucose Cotransporter 2 Inhibitor Use Among Patients With Diabetes in the US. JAMA Netw Open (2021) 4(4):e216139. doi: 10.1001/jamanetworkopen.2021.6139 33856475 PMC8050743

[3] Dennis JM Henley WE McGovern AP Farmer AJ Sattar N Holman RR. Time Trends in Prescribing of Type 2 Diabetes Drugs, Glycaemic Response and Risk Factors: A Retrospective Analysis of Primary Care Data, 2010-2017. Diabetes Obes Metab (2019) 21:1576–84. doi: 10.1111/dom.13687 PMC6618851 30828962

[4] Kim J Park S Kim H Je NK. National Trends in Metformin-Based Combination Therapy of Oral Hypoglycaemic Agents for Type 2 Diabetes Mellitus. Eur J Clin Pharmacol (2019) 75(12):1723–30. doi: 10.1007/s00228-019-02751-9 31475315

[5] Lee G Oh SW Hwang SS Yoon JW Kang S Joh HK. Comparative Effectiveness of Oral Antidiabetic Drugs in Preventing Cardiovascular Mortality and Morbidity: A Network Meta-Analysis. PloS One (2017) 12(5):e0177646. doi: 10.1371/journal.pone.0177646 28542373 PMC5444626

[6] Zheng SL Roddick AJ Aghar-Jaffar R Shun-Shin MJ Francis D Oliver N. Association Between Use of Sodium-Glucose Cotransporter 2 Inhibitors, Glucagon-Like Peptide 1 Agonists, and Dipeptidyl Peptidase 4 Inhibitors With All-Cause Mortality in Patients With Type 2 Diabetes: A Systematic Review and Meta-Analysis. JAMA (2018) 319(15):1580–91. doi: 10.1001/jama.2018.3024 PMC5933330 29677303

[7] Patorno E Goldfine AB Schneeweiss S Everett BM Glynn RJ Liu J. Cardiovascular Outcomes Associated With Canagliflozin Versus Other non-Gliflozin Antidiabetic Drugs: Population Based Cohort Study. BMJ (2018) 360:k119. doi: 10.1136/bmj.k119 29437648 PMC5799855

[8] Persson F Nyström T Jørgensen ME Carstensen B Gulseth HL Thuresson M. Dapagliflozin Is Associated With Lower Risk of Cardiovascular Events and All-Cause Mortality in People With Type 2 Diabetes (CVD-REAL Nordic) When Compared With Dipeptidyl Peptidase-4 Inhibitor Therapy: A Multinational Observational Study. Diabetes Obes Metab (2018) 20:344–51. doi: 10.1111/dom.13077 PMC5811811 28771923

[9] Pasternak B Ueda P Eliasson B Svensson A Franzén S Gudbjörnsdottir S. Use of Sodium Glucose Cotransporter 2 Inhibitors and Risk of Major Cardiovascular Events and Heart Failure: Scandinavian Register Based Cohort Study. BMJ (2019) 366:l4772. doi: 10.1136/bmj.l4772 31467044 PMC6713906

[10] Filion KB Lix LM Yu OH Dell' Aniello S Douros A Shah BR Canadian Network for Observational Drug Effect Studies (CNODES) Investigators. Sodium Glucose Cotransporter 2 Inhibitors and Risk of Major Adverse Cardiovascular Events: Multi-Database Retrospective Cohort Study. BMJ (2020) 370:m3342. doi: 10.1136/bmj.m3342 32967856 PMC8009082

[11] Kohsaka S Lam CSP Kim DJ Cavender MA Norhammar A Jørgensen ME. CVD-REAL 2 Investigators and Study Group. Risk of Cardiovascular Events and Death Associated With Initiation of SGLT2 Inhibitors Compared With DPP-4 Inhibitors: An Analysis From the CVD-REAL 2 Multinational Cohort Study. Lancet Diabetes Endocrinol (2020) 8:606–15. doi: 10.1016/S2213-8587(20)30130-3 32559476

[12] Ma RC Chan JC. Type 2 Diabetes in East Asians: Similarities and Differences With Populations in Europe and the United States. Ann N Y Acad Sci (2013) 1281(1):64–91. doi: 10.1111/nyas.12098 23551121 PMC3708105

[13] Ma RCW. Epidemiology of Diabetes and Diabetic Complications in China. Diabetologia (2018) 61(6):1249–60. doi: 10.1007/s00125-018-4557-7 29392352

[14] Seino Y Kim DJ Yabe D Tan EC Chung WJ Ha KH EMPRISE East Asia study group. Cardiovascular and Renal Effectiveness of Empagliflozin in Routine Care in East Asia: Results From the EMPRISE East Asia Study. Endocrinol Diabetes Metab (2021) 4:e00183. doi: 10.1002/edm2.183 33532619 PMC7831226

[15] Birkeland KI Bodegard J Banerjee A Kim DJ Norhammar A Eriksson JW. Lower Cardiorenal Risk With Sodium-Glucose Cotransporter-2 Inhibitors Versus Dipeptidyl Peptidase-4 Inhibitors in Patients With Type 2 Diabetes Without Cardiovascular and Renal Diseases: A Large Multinational Observational Study. Diabetes Obes Metab (2021) 23:75–85. doi: 10.1111/dom.14189 32893440 PMC7756303

[16] Kohsaka S Takeda M Bodegård J Thuresson M Kosiborod M Yajima T. Sodium-Glucose Cotransporter 2 Inhibitors Compared With Other Glucose-Lowering Drugs in Japan: Subanalyses of the CVD-REAL 2 Study. J Diabetes Investig (2021) 12:67–73. doi: 10.1111/jdi.13321 PMC7779275 32530554

[17] Komuro I Kadowaki T Bodegård J Thuresson M Okami S Yajima T. Lower Heart Failure and Chronic Kidney Disease Risks Associated With Sodium-Glucose Cotransporter-2 Inhibitor Use in Japanese Type 2 Diabetes Patients Without Established Cardiovascular and Renal Diseases. Diabetes Obes Metab (2021) 23(Suppl 2):19–27. doi: 10.1111/dom.14119 33835641

[18] Ling AW Chan CC Chen SW Kao YW Huang CY Chan YH. The Risk of New-Onset Atrial Fibrillation in Patients With Type 2 Diabetes Mellitus Treated With Sodium Glucose Cotransporter 2 Inhibitors Versus Dipeptidyl Peptidase-4 Inhibitors. Cardiovasc Diabetol (2020) 19:188. doi: 10.1186/s12933-020-01162-w 33158436 PMC7648323

[19] Seong JM Kim JJ Kim HJ Sohn HS. Comparison of Heart Failure Risk and Medical Costs Between Patients With Type 2 Diabetes Mellitus Treated With Dapagliflozin and Dipeptidyl Peptidase-4 Inhibitors: A Nationwide Population-Based Cohort Study. Cardiovasc Diabetol (2020) 19(1):95. doi: 10.1186/s12933-020-01060-1 32571319 PMC7310428

[20] Kim YG Han SJ Kim DJ Lee KW Kim HJ. Association Between Sodium Glucose Co-Transporter 2 Inhibitors and a Reduced Risk of Heart Failure in Patients With Type 2 Diabetes Mellitus: A Real-World Nationwide Population-Based Cohort Study. Cardiovasc Diabetol (2018) 17(1):91. doi: 10.1186/s12933-018-0737-5 29935543 PMC6015464

[21] Han SJ Ha KH Lee N Kim DJ. Effectiveness and Safety of Sodium-Glucose Co-Transporter-2 Inhibitors Compared With Dipeptidyl Peptidase-4 Inhibitors in Older Adults With Type 2 Diabetes: A Nationwide Population-Based Study. Diabetes Obes Metab (2021) 23(3):682–91. doi: 10.1111/dom.14261 PMC7898287 33236515

[22] Lee HF Chen SW Liu JR Li PR Wu LS Chang SH. Major Adverse Cardiovascular and Limb Events in Patients With Diabetes and Concomitant Peripheral Artery Disease Treated With Sodium Glucose Cotransporter 2 Inhibitor Versus Dipeptidyl Peptidase-4 Inhibitor. Cardiovasc Diabetol (2020) 19(1):160. doi: 10.1186/s12933-020-01118-0 32998736 PMC7528264

[23] Hsieh CY Su CC Shao SC Sung SF Lin SJ Kao YH. Taiwan’s National Health Insurance Research Database: Past and Future. Clin Epidemiol (2019) 11:349–58. doi: 10.2147/CLEP.S196293 PMC6509937 31118821

[24] Lund JL Richardson DB Stürmer T. The Active Comparator, New User Study Design in Pharmacoepidemiology: Historical Foundations and Contemporary Application. Curr Epidemiol Rep (2015) 2(4):221–8. doi: 10.1007/s40471-015-0053-5 PMC4778958 26954351

[25] Yang CT Lin WH Li LJ Ou HT Kuo S. Association of Renal and Cardiovascular Safety With DPP-4 Inhibitors vs. Sulfonylureas in Patients With Type 2 Diabetes and Advanced Chronic Kidney Disease. Clin Pharmacol Ther (2021) 110(2):464–72. doi: 10.1002/cpt.2262 33866549

[26] Parsons L. Reducing Bias in a Propensity Score Matched-Pai Sample Using Greedy Matching Techniques. Available at: https://support.sas.com/resources/papers/proceedings/proceedings/sugi26/p214-26.pdf (Accessed 10 June 2021).

[27] Yang D Dalton JE. A Unified Approach to Measuring the Effect Size Between Two Groups Using SAS. SAS Global Forum 2012: Statistics and Data Analysis, Paper 335-2012 . Available at: https://support.sas.com/resources/papers/proceedings12/335-2012.pdf (Accessed 10 June 2021).

[28] Austin PC Lee DS Fine JP. Introduction to the Analysis of Survival Data in the Presence of Competing Risks. Circulation (2016) 133:601–9. doi: 10.1161/CIRCULATIONAHA.115.017719 PMC4741409 26858290

[29] Liberati A Altman DG Tetzlaff J Mulrow C Gøtzsche PC Ioannidis JP. The PRISMA Statement for Reporting Systematic Reviews and Meta-Analyses of Studies That Evaluate Healthcare Interventions: Explanation and Elaboration. BMJ (2009) 339:b2700. doi: 10.1136/bmj.b2700 19622552 PMC2714672

[30] Kim YG Jeon JY Han SJ Kim DJ Lee KW Kim HJ. Sodium-Glucose Co-Transporter-2 Inhibitors and the Risk of Ketoacidosis in Patients With Type 2 Diabetes Mellitus: A Nationwide Population-Based Cohort Study. Diabetes Obes Metab (2018) 20(8):1852–8. doi: 10.1111/dom.13297 29569427

[31] Li WJ Chen XQ Xu LL Li YQ Luo BH. SGLT2 Inhibitors and Atrial Fibrillation in Type 2 Diabetes: A Systematic Review With Meta-Analysis of 16 Randomized Controlled Trials. Cardiovasc Diabetol (2020) 19(1):130. doi: 10.1186/s12933-020-01105-5 32847602 PMC7448518

[32] Hayes AJ Leal J Gray AM Holman RR Clarke PM. UKPDS Outcomes Model 2: A New Version of a Model to Simulate Lifetime Health Outcomes of Patients With Type 2 Diabetes Mellitus Using Data From the 30 Year United Kingdom Prospective Diabetes Study: UKPDS 82. Diabetologia (2013) 56(9):1925–33. doi: 10.1007/s00125-013-2940-y 23793713

[33] Lip GYH Brechin CM Lane DA. The Global Burden of Atrial Fibrillation and Stroke: A Systematic Review of the Epidemiology of Atrial Fibrillation in Regions Outside North America and Europe. Chest (2012) 142(6):1489–98. doi: 10.1378/chest.11-2888 22459778

[34] Chao TF Wang KL Liu CJ Lin YJ Chang SL Lo LW. Age Threshold for Increased Stroke Risk Among Patients With Atrial Fibrillation: A Nationwide Cohort Study From Taiwan. J Am Coll Cardiol (2015) 66(12):1339–47. doi: 10.1016/j.jacc.2015.07.026 26383720

[35] Cha MJ Choi EK Han KD Lee SR Lim WH Oh S. Effectiveness and Safety of Non-Vitamin K Antagonist Oral Anticoagulants in Asian Patients With Atrial Fibrillation. Stroke (2017) 48(11):3040–8. doi: 10.1161/STROKEAHA.117.018773 28974629

[36] Wang KL Lip GY Lin SJ Chiang CE. Non-Vitamin K Antagonist Oral Anticoagulants for Stroke Prevention in Asian Patients With Nonvalvular Atrial Fibrillation: Meta-Analysis. Stroke (2015) 46:2555–61. doi: 10.1161/STROKEAHA.115.009947 PMC4542566 26304863

[37] Kim J Thayabaranathan T Donnan GA Howard G Howard VJ Rothwell PM. Global Stroke Statistics 2019. Int J Stroke (2020) 15(8):819–38. doi: 10.1177/1747493020909545 32146867

[38] Kim AS Johnston SC. Global Variation in the Relative Burden of Stroke and Ischemic Heart Disease. Circulation (2011) 124:314–23. doi: 10.1161/CIRCULATIONAHA.111.018820 21730306

[39] Pasternak B Wintzell V Melbye M Eliasson B Svensson AM Franzén S. Use of Sodium-Glucose Co-Transporter 2 Inhibitors and Risk of Serious Renal Events: Scandinavian Cohort Study. BMJ (2020) 369:m1186. doi: 10.1136/bmj.m1186 32349963 PMC7188014

[40] Xie Y Bowe B Gibson AK McGill JB Maddukuri G Yan Y. Comparative Effectiveness of SGLT2 Inhibitors, GLP-1 Receptor Agonists, DPP-4 Inhibitors, and Sulfonylureas on Risk of Kidney Outcomes: Emulation of a Target Trial Using Health Care Databases. Diabetes Care (2020) 43(11):2859–69. doi: 10.2337/dc20-1890 32938746

[41] Adimadhyam S Lee TA Calip GS Smith Marsh DE Layden BT Schumock GT. Risk of Amputations Associated With SGLT2 Inhibitors Compared to DPP-4 Inhibitors: A Propensity-Matched Cohort Study. Diabetes Obes Metab (2018) 20(12):2792–9. doi: 10.1111/dom.13459 29971914

[42] Chang HY Singh S Mansour O Baksh S Alexander GC. Association Between Sodium-Glucose Cotransporter 2 Inhibitors and Lower Extremity Amputation Among Patients With Type 2 Diabetes. JAMA Intern Med (2018) 178(9):1190–8. doi: 10.1001/jamainternmed.2018.3034 PMC6142968 30105373

[43] Yu OHY Dell'Aniello S Shah BR Brunetti VC Daigle JM Fralick M Canadian Network for Observational Drug Effect Studies (CNODES) Investigators. Sodium-Glucose Cotransporter 2 Inhibitors and the Risk of Below-Knee Amputation: A Multicenter Observational Study. Diabetes Care (2020) 43(10):2444–52. doi: 10.2337/dc20-0267 32759360

[44] Unwin N. Epidemiology of Lower Extremity Amputation in Centres in Europe, North America and East Asia. Br J Surg (2000) 87(3):328–37. doi: 10.1046/j.1365-2168.2000.01344.x 10718803

[45] Young BA Maynard C Reiber G Boyko EJ. Effects of Ethnicity and Nephropathy on Lower-Extremity Amputation Risk Among Diabetic Veterans. Diabetes Care (2003) 26(2):495–501. doi: 10.2337/diacare.26.2.495 12547888

[46] Abbott CA Garrow AP Carrington AL Morris J Van Ross ER Boulton AJ. Foot Ulcer Risk Is Lower in South-Asian and African-Caribbean Compared With European Diabetic Patients in the U.K.: The North-West Diabetes Foot Care Study. Diabetes Care (2005) 28(8):1869–75. doi: 10.2337/diacare.28.8.1869 16043725

[47] Chaturvedi N Abbott CA Whalley A Widdows P Leggetter SY Boulton AJ. Risk of Diabetes-Related Amputation in South Asians vs. Europeans in the UK. Diabetes Med (2002) 19(2):99–104. doi: 10.1046/j.1464-5491.2002.00583.x 11874424

