# Supplementary material for: Cardiovascular Benefits With Favorable Renal, Amputation and Hypoglycemic Outcomes of SGLT-2 Inhibitors in Type 2 Diabetes From the Asian Perspective: A Population-Based Cohort Study and Systematic Review
Source: Front Endocrinol (Lausanne). 2022 Mar 7;13:836365. doi: 10.3389/fendo.2022.836365 (PMC8940301; doi:10.3389/fendo.2022.836365)
Supplement: Supplementary file 1 [file DataSheet_1.pdf]

Online-only Supplements

Supplementary Table 1. Operational definitions of baseline characteristics defined by ICD-9-CM and ICD-10-CM codes and source of data for measurement
Supplementary Table 2: Operational definitions of study outcomes defined by ICD-9-CM and ICD-10-CM codes and source of data for measurement
Supplementary Table 3: Search strategies in a systematic review for effects of SGLT2is versus DPP4is on cardiovascular diseases and safety outcomes in Asian patients with type 2 diabetes
Supplementary Table 4: Event rates and hazard ratios of clinical outcomes associated with use of SGLT2is versus DPP4is (as-treated analyses)
Supplementary Table 5: Real-world evidence of clinical outcomes of SGLT2is versus DPP4is in Asian patients with type 2 diabetes
Supplementary Figure 1: Flowchart of study cohort selection
Supplementary Figure 2: Kernel density curves of propensity score distributions for two study groups (SGLT2is and DPP4is) before and after matching
Supplementary Figure 3: Flowchart of study selection in a systematic review for clinical outcomes of SGLT2is versus DPP4is in Asian patients with type 2 diabetes

Supplementary Table 1. Operational definitions of baseline characteristics defined by ICD-9-CM and ICD-10-CM codes and source of data for measurement

**Baseline characteristics**	**ICD-9-CM diagnosis or procedure codes**	**ICD-10-CM diagnosis or procedure codes**	**Data files in NHIRD**
Nephropathy[1,2]	250.4, 580, 581, 582, 583, 585, 586, 593.9	E08.2, E09.2, E10.2, E11.2, E13.2, N03, N04, N05, N17, N18, N19, T82.4, Z49, Z99.2	Outpatient, inpatient, and emergency department
Neuropathy[1,2]	250.6, 354, 355, 356.9, 357.2, 358.1, 713.5, 951.0, 951.1, 951.3	E08.4, E09.4, E10.4, E11.4, E13.4, G60.9, G73.3, H49.0, H49.1, H49.2	
Retinopathy[1,2]	250.5, 362.01, 362.02, 361, 362.1, 362.81-362.83, 362.53, 369, 379.23	E08.3, E09.3, E10.3, E11.3, E13.3, H33.0, H54.0, H54.4, H43.1	
Peripheral vascular disease[1,2]	250.7, 442.3, 443.81, 443.9, 892.1, 443.9, 444.22, 785.4, 0.4, 707.1	E08.5, E09.5, E10.5, E11.5, E13.5, I72.4, I73.9, I74.3, I70.23-I70.26, A48.0, L97, L98.4, E08.621, E08.622, E09.621, E09.622, E10.621, E10.622, E11.621, E11.622, E13.621, E13.622	
Cerebrovascular disease[3,4]	433, 434, 430, 431, 432, 435, V1254	I63, I60, I61, I62, G45	
Cardiovascular disease[3-5]	428, 410, 411, 413, 414, V4581, V4582, 4292, 426, 427, 78551, V1253	I50, I21, I25, I2510, I44, I45, I46, I47, I48, I49, R57, Z8674	
Heart failure[5]	428	I50	
Acute myocardial infarction[4,5]	410	I21	
Ischemic heart disease[6,7]	411, 413, 414, V4581, V4582	I25	
Diabetic ketoacidosis[1,2]	250.1, 250.2, 250.3	E08.1, E09.1, E10.1, E11.1, E13.1, E08.641, E09.641, E10.641, E11.641, E13.641	
Hypoglycemia[8,9]	2508, 2510, 2511, 2512, 9623	E15, E160, E161, E162, E1164, T38.3 (except T38.3X6)	

Abbreviations: ICD-9-CM, International Classification of Diseases ninth edition Clinical Modification; ICD-10-CM, International Classification of Diseases tenth edition Clinical Modification; NHIRD, National Health Insurance Research Database.

References

1. *Am J Manag Care* 2012;18(11):721-726.
2. *Am J Manag Care* 2019;25(2):e45-e49.
3. *Diabetes Care* 2018;41(5):917-928.
4. *J Epidemiol* 2014;24(6):500-507.
5. *Diabetes Care* 2018;41(5):917-28.
6. *Acta Psychiatr Scand* 2019;140(3):265-274.
7. *J Am Heart Assoc* 2017;6(2).
8. *BMC Endocr Disord* 2008;8:4.
9. *Diabetes Care* 2019;42(4):e58-59.

Supplementary Table 2. Operational definitions of study outcomes defined by ICD-9-CM and ICD-10-CM codes and source of data for measurement

Clinical outcomes	ICD-9-CM diagnosis or procedure codes†	ICD-10-CM diagnosis or procedure codes†	Data files in NHIRD
Myocardial infarction[1]	410	I21	Inpatient and emergency department
Stroke[2,3]	430, 431-434	I60-I63	Inpatient and emergency department
Heart failure[4]	428	I50	Inpatient and emergency department
Chronic kidney disease	585	N18	Outpatient department
Amputation[5]	84.1	0Y67, 0Y68, 0Y6C, 0Y6D, 0Y6F, 0Y6G, 0Y6H, 0Y6J, 0Y6M, 0Y6N, 0Y6P, 0Y6Q, 0Y6R, 0Y6S, 0Y6T, 0Y6U, 0Y6V, 0Y6W, 0Y6X, 0Y6Y	Inpatient department
Hospitalized hypoglycemia[6,7]	250.8, 251.0-251.2, 962.3	E15, E160-E162, E11.64, T38.3 (except for T38.3X6)	Inpatient and emergency department

Abbreviations: ICD-9-CM, International Classification of Diseases ninth edition Clinical Modification; ICD-10-CM, International Classification of Diseases tenth edition Clinical Modification; NHIRD, National Health Insurance Research Database.

†Clinical outcomes of myocardial infarction, stroke, heart failure, chronic kidney disease, and hospitalized hypoglycemia were identified by ICD-9-CM and ICD-10-CM diagnosis codes, and amputation was identified by ICD-9-CM and ICD-10-CM procedure codes.

References

1. *J Epidemiol* 2014;24(6):500-7.
2. *Pharmacoepidemiology and Drug Safety* 2011;20(3):236-42.
3. *J Formos Med Assoc* 2015;114(3):254-9.
4. *Diabetes Care* 2018;41(5):917-28.
5. *Cardiovasc Diabetol* 2020;19(1):160.
6. *BMC Endocr Disord* 2008;8:4.
7. *Diabetes Care* 2019;42(4):e58-e59.

Supplementary Table 3. Search strategies in a systematic review for effects of SGLT2is versus DPP4is on cardiovascular diseases and safety outcomes in Asian patients with type 2 diabetes

We searched for all available studies in PubMed and Embase from the inception of the databases to May 13, 2021, that reported the treatment effects of sodium glucose cotransporter 2 inhibitors (SGLT2is) versus dipeptidyl peptidase 4 inhibitors (DPP4is). The search strategies using the framework of "Population Intervention Comparison Outcome (PICO)" and keywords are listed in the following table. 856 and 689 articles were identified in PubMed and Embase, respectively. After the removal of duplicates, conference abstracts, studies that did not compare the effects of SGLT2is versus DPP4is on clinical outcomes, unmatched PICO, and studies that lacked full-text English articles, a total of 59 studies were selected for the assessment of eligibility. Among these 61 studies, only 11 studies involved Asian populations, and thus were included for the full-text review.

Search strategies for literature review in the framework of Population Intervention Comparison Outcome (PICO)

#	Keywords
	**Population**
1	Type 2 diabetes
	**Intervention**
2	Sodium glucose cotransporter 2 inhibitor
3	SGLT2 inhibitor
4	Empagliflozin
5	Dapagliflozin
6	Canagliflozin
7	Ertugliflozin
8	Ipragliflozin
9	Luseogliflozin
10	Remogliflozin
11	Sergliflozin
12	Sotagliflozin
13	Tofogliflozin
	**Comparison**
14	Dipeptidyl peptidase 4 inhibitor
15	DPP4 inhibitor
16	Sitagliptin
17	Saxagliptin
18	Vildagliptin
19	Linagliptin
20	Alogliptin
21	Gemigliptin
22	Anagliptin
23	Teneligliptin

24	Trelagliptin
25	Omarigliptin
26	Evogliptin
27	Gosogliptin
	**Outcome**
28	Cardiovascular disease
29	Cardiovascular outcome
30	Heart disease
31	Heart outcome
32	Macrovascular disease
33	Macrovascular outcome
34	Heart failure
35	Stroke
36	Myocardial infarction
37	Major adverse cardiovascular event
38	Major adverse cardiac event
39	Kidney
40	Renal
41	Amputation
42	Hypoglycemia
43	Mortality
44	Death
45	Effectiveness
46	Safety
47	Adverse
	**Search strategy**: 1 AND (2 OR 3 OR 4 OR 5 OR 6 OR 7 OR 8 OR 9 OR 10 OR 11 OR 12 OR 13) AND (14 OR 15 OR 16 OR 17 OR 18 OR 19 OR 20 OR 21 OR 22 OR 23 OR 24 OR 25 OR 26 OR 27) AND (28 OR 29 OR 30 OR 31 OR 32 OR 33 OR 34 OR 35 OR 36 OR 37 OR 38 OR 39 OR 40 OR 41 OR 42 OR 43 OR 44 OR 45 OR 46 OR 47)

Supplementary Table 4. Event rates and hazard ratios of clinical outcomes associated with use of SGLT2is versus DPP4is (as-treated analyses)

	SGLT2is (n=21,329)		DPP4is (n=21,329)		
	Number of events	Event rate (events/100 pys)	Number of events	Event rate (events/100 pys)	SDHR of SGLT2is versus DPP4is (95% CI)
**Primary outcomes**					
HFF	340	1.17	658	2.30	0.52 (0.45, 0.59)
3P-MACE†	389	1.32	641	2.19	0.63 (0.55, 0.71)
**Secondary outcomes**					
4P-MACE‡	660	2.24	1,144	3.91	0.59 (0.54, 0.65)
Myocardial infarction	114	0.39	188	0.65	0.61 (0.48, 0.77)
Stroke	252	0.86	428	1.49	0.59 (0.50, 0.69)
All-cause death§	244	0.83	431	1.49	0.56 (0.48, 0.65)
Chronic kidney disease	979	3.59	2,003	8.01	0.46 (0.43, 0.50)
Amputation	32	0.11	55	0.19	0.58 (0.38, 0.90)
Hospitalized hypoglycemia	185	0.63	346	1.20	0.53 (0.45, 0.64)

Abbreviations: SGLT2is, sodium glucose cotransporter 2 inhibitors; DPP4is, dipeptidyl peptidase 4 inhibitors; pys, person-years; SDHR, subdistribution hazard ratio; HHF, hospitalization for heart failure; MACE, major adverse cardiovascular event.

†3P-MACE included non-fatal myocardial infarction, non-fatal stroke, or cardiovascular death.

‡4P-MACE included heart failure, non-fatal myocardial infarction, non-fatal stroke, or cardiovascular death.

§Hazard ratio of all-cause death was estimated using the Cox proportional hazard model instead of subdistribution hazard model.

Supplementary Table 5. Real-world evidence of clinical outcomes of SGLT2is versus DPP4is in Asian patients with type 2 diabetes

Author, year [reference]	Database and country	Target population and study design	Sample size (SGLT2is vs. DPP4is)	Study outcomes	Event rates, events per 100 pys (SGLT2is vs. DPP4is)	Hazard ratios of SGLT2is vs. DPP4is (95% CIs)
**Multinational studies that involved Asian countries**						
Seino, 2021 [14][†]	MDV database 2014-2018, Japan; NHIS 2016-2017, Korea; NHIRD 2016-2017, Taiwan	• T2D<br>• INU cohort design and 1:1 PSM for baseline adjustment (PSM was performed for each country)	Before PSM (patients):<br>- Japan: 432,054<br>- Korea: 276,983<br>- Taiwan: 329,065<br><br>After PSM (pairs):<br>- Japan: 5,592<br>- Korea: 9,072<br>- Taiwan: 14,048	HHF	Overall: 2.53 vs. 3.16<br>Japan: 6.68 vs. 8.82<br>Korea: 1.53 vs. 2.07<br>Taiwan: 1.64 vs. 1.87	Overall: **0.82 (0.71-0.94)**<br>Japan: **0.80 (0.66-0.98)**<br>Korea: 0.74 (0.55-1.00)<br>Taiwan: 0.90 (0.70-1.17)
				All-cause death	Overall: 0.77 vs. 1.22<br>Japan: 0.72 vs. 1.19<br>Korea: 0.36 vs. 0.68<br>Taiwan: 1.10 vs. 1.63	Overall: **0.64 (0.50-0.81)**<br>Japan: 0.63 (0.35-1.10)<br>Korea: **0.52 (0.29-0.93)**<br>Taiwan: **0.68 (0.50-0.91)**
				ESRD	Overall: 0.18 vs. 0.55<br>Japan: 0.22 vs. 0.68<br>Korea: 0 vs. 0.19<br>Taiwan: 0.30 vs. 0.77	Overall: **0.37 (0.24-0.58)**<br>Japan: **0.33 (0.13-0.85)**<br>Korea: not available<br>Taiwan: **0.38 (0.23-0.64)**
Birkeland, 2021 [15][‡]	Medical records from Europe and Asia<br>- MDV database, Japan<br>- NHIS, Korea	• T2D with no history of CVD and CKD<br>• INU cohort design and 1:1 PSM for baseline adjustment	Before PSM: 116,093 vs. 890,484 patients<br>After PSM: 105,130 pairs	HF	Overall: 2.9 vs. 4.1<br>Japan: 0.28 vs. 0.46<br>Korea: 0.40 vs. 0.55	Overall: **0.71 (0.59-0.86)**<br>Japan: **0.63 (0.43-0.92)**<br>Korea: **0.73 (0.57-0.93)**
				Stroke	Overall: 4.4 vs. 4.8<br>Japan: 0.28 vs. 0.52<br>Korea: 0.47 vs. 0.47	Overall: 0.87 (0.69-1.09)<br>Japan: **0.54 (0.37-0.78)**<br>Korea: 0.98 (0.77-1.25)
				MI	Overall: 3.0 vs. 3.2<br>Japan: 0.09 vs. 0.06<br>Korea: 0.21 vs. 0.28	Overall: 0.94 (0.80-1.11)<br>Japan: 1.56 (0.65-3.72)<br>Korea: 0.74 (0.53-1.04)

				All-cause death	Overall: 4.3 vs. 6.3<br>Japan: 0.44 vs. 0.72<br>Korea: 0.28 vs. 0.31	Overall: **0.67 (0.59-0.77)**<br>Japan: **0.62 (0.46-0.84)**<br>Korea: 0.88 (0.65-1.20)
				CKD	Overall: 3.7 vs. 7.9<br>Japan: 0.28 vs. 1.28<br>Korea: 0.44 vs. 0.62	Overall: **0.44 (0.28-0.69)**<br>Japan: **0.23 (0.17-0.32)**<br>Korea: **0.69 (0.55-0.87)**
				Cardiorenal diseases	Overall: 6.3 vs. 11.5<br>Japan: 0.57 vs. 1.71<br>Korea: 0.80 vs. 1.10	Overall: **0.56 (0.42-0.74)**<br>Japan: **0.35 (0.27-0.45)**<br>Korea: **0.72 (0.61-0.86)**
Kohsaka, 2020 [11][§]	CVD-REAL 2 2012-2017, Asia-Pacific, Middle East, Europe, North America	• T2D<br>• INU cohort design and 1:1 PSM for baseline adjustment	Before PSM (patients):<br>- Total: 230,721 vs. 2,182,477<br>- Korea: 69,805 vs. 980,429<br>- Japan: 11,071 vs. 133,979<br>- Taiwan: 16,255 vs. 646,704<br>- Singapore: 2,828 vs. 1,770<br><br>After PSM: 193,124 pairs	HHF	Overall: 0.79 vs. 1.07<br>Korea: 1.24 vs. 1.45<br>Japan: 0.61 vs. 0.77<br>Taiwan: 0.55 vs. 0.76<br>Singapore: 1.52 vs. 1.71	**Overall: 0.69 (0.61-0.77)**<br>Korea: **0.85 (0.78-0.93)**<br>Japan: 0.79 (0.57-1.10)<br>Taiwan: 0.72 (0.41-1.27)<br>Singapore: 0.87 (0.38-2.01)
				Stroke	Overall: 0.82 vs. 0.99<br>Korea: 1.48 vs. 1.76<br>Japan: 0.26 vs. 0.42<br>Taiwan: 0.94 vs. 1.00<br>Singapore: 0.15 vs. 0.70	Overall: **0.85 (0.77-0.93)**<br>Korea: **0.83 (0.77-0.91)**<br>Japan: 0.64 (0.39-1.04)<br>Taiwan: 0.94 (0.60-1.49)<br>Singapore: 0.20 (0.02-1.71)
				MI	Overall: 0.52 vs. 0.58<br>Korea: 0.47 vs. 0.51<br>Japan: 0.11 vs. 0.10<br>Taiwan: 0.26 vs. 0.50<br>Singapore: 1.37 vs. 1.54	Overall: **0.88 (0.80-0.98)**<br>Korea: 0.92 (0.79-1.07)<br>Japan: 1.09 (0.46-2.57)<br>Taiwan: 0.52 (0.24-1.13)<br>Singapore:0.89 (0.37-2.15)
				All-cause death	Overall: 0.84 vs. 1.37<br>Korea: 0.80 vs. 1.04<br>Japan: 0.47 vs. 0.91<br>Taiwan: 1.30 vs. 3.87	Overall: **0.59 (0.52-0.67)**<br>Korea: **0.77 (0.69-0.86)**<br>Japan: **0.52 (0.37-0.74)**<br>Taiwan: **0.34 (0.24-0.46)**

					Singapore: 0.90 vs. 1.12	Singapore: 0.80 (0.28-2.31)
				HHF or all-cause death	Overall: 1.56 vs. 2.27<br>Korea: 1.92 vs. 2.31<br>Japan: 1.03 vs. 1.59<br>Taiwan: 1.85 vs. 4.53<br>Singapore: 2.12 vs. 2.56	Overall: **0.64 (0.57-0.72)**<br>Korea: **0.83 (0.77-0.89)**<br>Japan: **0.65 (0.51-0.83)**<br>Taiwan: **0.41 (0.31-0.54)**<br>Singapore: 0.82 (0.41-1.65)
**Single-country studies conducted in Asia**						
Kohsaka, 2021 [16]	CVD-REAL 2 2012-2017, Japan	• T2D<br>• INU cohort design and 1:1 PSM for baseline adjustment	Before PSM: 11,071 vs. 133,979 patients<br>After PSM: 9,876 pairs	HHF	0.61 vs. 0.77	0.79 (0.57-1.10)
				Stroke	0.26 vs. 0.42	0.64 (0.39-1.04)
				MI	0.11 vs. 0.10	1.09 (0.46-2.57)
				Death	0.47 vs. 0.91	**0.52 (0.37-0.74)**
				HHF or death	1.03 vs. 1.59	**0.65 (0.51-0.83)**
Han, 2021 [21]	NHIS 2014-2016, Korea	• T2D aged ≧65 years<br>• INU cohort design and 1:1 PSM for baseline adjustment	Before PSM: 15,703 vs. 392,803 patients<br>After PSM: 15,699 pairs	HHF	2.82 vs. 3.28	**0.86 (0.76-0.97)**
				Stroke	3.30 vs. 3.82	**0.86 (0.77-0.97)**
				MI	0.94 vs. 0.98	0.95 (0.77-1.19)
				All-cause death	2.30 vs. 2.69	**0.85 (0.75-0.98)**
				HHF or all-cause death	4.72 vs. 5.51	**0.86 (0.78-0.94)**
				DKA	0.26 vs. 0.27	0.96 (0.63-1.46)
				Bone fracture	8.87 vs. 9.31	0.95 (0.88-1.02)
				Severe hypoglycemia	2.40 vs. 2.56	0.93 (0.81-1.07)
				Genital infection	9.93 vs. 4.01	**2.44 (2.22-2.67)**
				UTI	20.37 vs. 19.17	1.05 (1.00-1.11)
Komuro, 2020 [17]	MDV database 2014-2018, Japan	• T2D aged ≥ 18 years<br>• CVD- and CKD-free patients<br>• INU cohort design and 1:1 PSM for baseline	Before PSM: 19,642 vs. 161,726 patients<br>After PSM: 17,232 pairs	HHF	0.28 vs. 0.32	0.86 (0.63-1.18)
				Stroke	0.25 vs. 0.46	**0.54 (0.40-0.73)**
				MI	0.08 vs. 0.04	1.97 (0.95-4.06)
				All-cause death	0.50 vs. 0.78	**0.64 (0.51-0.80)**
				CKD	0.26 vs. 0.92	**0.29 (0.22-0.38)**

		adjustment		Cardiorenal disease	0.54 vs. 1.21	**0.45 (0.37-0.55)**
Lee, 2020 [22]	NHIRD 2015-2017, Taiwan	• T2D with PAD<br>• INU cohort design and 1:1 PSM for baseline adjustment	Before PSM: 12,355 vs. 93,972 patients<br>After PSM: 11,431 pairs	HF	0.96 vs. 1.43	**0.66 (0.49-0.89)**
				Ischemic stroke	1.26 vs. 1.54	0.81 (0.62-1.06)
				MI	0.66 vs. 0.77	0.84 (0.58-1.23)
				CV death	0.91 vs. 1.33	**0.67 (0.49-0.90)**
				All-cause death	3.19 vs. 5.44	**0.58 (0.49-0.67)**
				Lower-limb ischemia	0.97 vs. 1.32	**0.73 (0.54-0.98)**
				Lower-limb amputation	0.54 vs. 1.23	**0.43 (0.30-0.62)**
				UTI	4.42 vs. 3.87	1.13 (0.96-1.32)
				Bone fracture	1.00 vs. 0.91	1.08 (0.78-1.50)
Ling, 2020 [18]	CGRD 2001-2018, Taiwan	• New-onset T2D with no history of AF before the diagnosis of T2D<br>• Cohort, IPTW for baseline adjustment	Before IPTW: 15,606 vs. 12,383 patients<br>After IPTW: 27,054 vs. 26,889 patients	New-onset AF	93 vs. 146 events during follow-up period (median: 1.48 and 1.05 years for SGLT2i and DPP4i users, respectively)	0.61 (0.50-0.73)
Seong, 2020 [19]	HIRA database 2014-2018, Korea	• T2D and no history of HHF, MI, and stroke in 8 weeks before index<br>• INU cohort design and PS-weighting for baseline adjustment	23,147 vs. 237,189 patients	HHF	AST analysis: 0.39 vs. 0.68<br>ITT analysis: 0.49 vs. 0.78	AST analysis: **0.58 (0.46-0.74)**<br>ITT analysis: **0.70 (0.60-0.82)**
Kim, 2018 [20]	HIRA database 2013-2017, Korea	• T2D and no history of HHF in 60 days before index<br>• INU cohort design and	Before PSM: 59,480 vs. 1,044,194 patients<br>After PSM:	HHF	0.83 vs. 1.13	**0.66 (0.58-0.75)**

		1:1 PSM for baseline adjustment	59,479 pairs			
Kim, 2018 [29]	NHIS 2013-2017, Korea	• T2D aged >19 years<br>• INU cohort design and 1:1 PSM for baseline adjustment	Before PSM: 56,326 vs. 974,179 patients<br>After PSM: 56,325 pairs	Hospitalization for DKA		
				In 30 days	0.25 vs. 0.30	0.85 (0.38-1.89)
				In 90 days	0.16 vs. 0.15	1.01 (0.53-1.94)
				In 180 days	0.11 vs. 0.12	0.90 (0.50-1.62)
				In 1 year	0.08 vs. 0.09	0.95 (0.56-1.61)
				In 3 years	0.06 vs. 0.07	0.96 (0.58-1.57)

Abbreviations: SGLT2i, sodium glucose cotransporter-2 inhibitor; DPP4i, dipeptidyl peptidase-4 inhibitor; pys, person-years; MDV, Medical Data Vision; NHIS, National Health Insurance Service; NHIRD, National Health Insurance Research Database; T2D, type 2 diabetes; INU, incident new user; PSM, propensity score matching; HHF, hospitalization for heart failure; ESRD, end-stage renal disease; HF, heart failure; MI, myocardial infarction; CKD; chronic kidney disease; CVD-REAL, Comparative Effectiveness of Cardiovascular Outcomes in New Users of SGLT-2 Inhibitors; DKA, diabetic ketoacidosis; UTI, urinary tract infection; AF, atrial fibrillation; IPTW, inverse-probability of treatment weighting; PAD, peripheral artery disease; AST, as-treated; ITT, intention-to-treat; HIRA, Health Insurance Review and Assessment Service.

Notes:

†Detailed information about the number of patients included in SGLT2i and DPP4i groups from each country was not provided.

‡Detailed information about the proportion of patients from different countries was not provided. This summarized table only presents the study findings (i.e., event rates and hazard ratios) of the overall study cohort and Japanese and Korean populations.

§Patients identified from South Korea, Japan, Taiwan, and Singapore accounted for around 36.1% (n=139,554), 5.1% (n=19,752), 6.0% (n=23,008), and 0.28% (n=1,100) of the propensity score-matched population in the CVD-REAL 2 analysis (i.e., 193,124 pairs of SGLT2i and DPP4i users), respectively. This summarized table only presents the study findings (i.e., event rates and hazard ratios) of Asia populations.

Supplementary Figure 1. Flowchart of study cohort selection

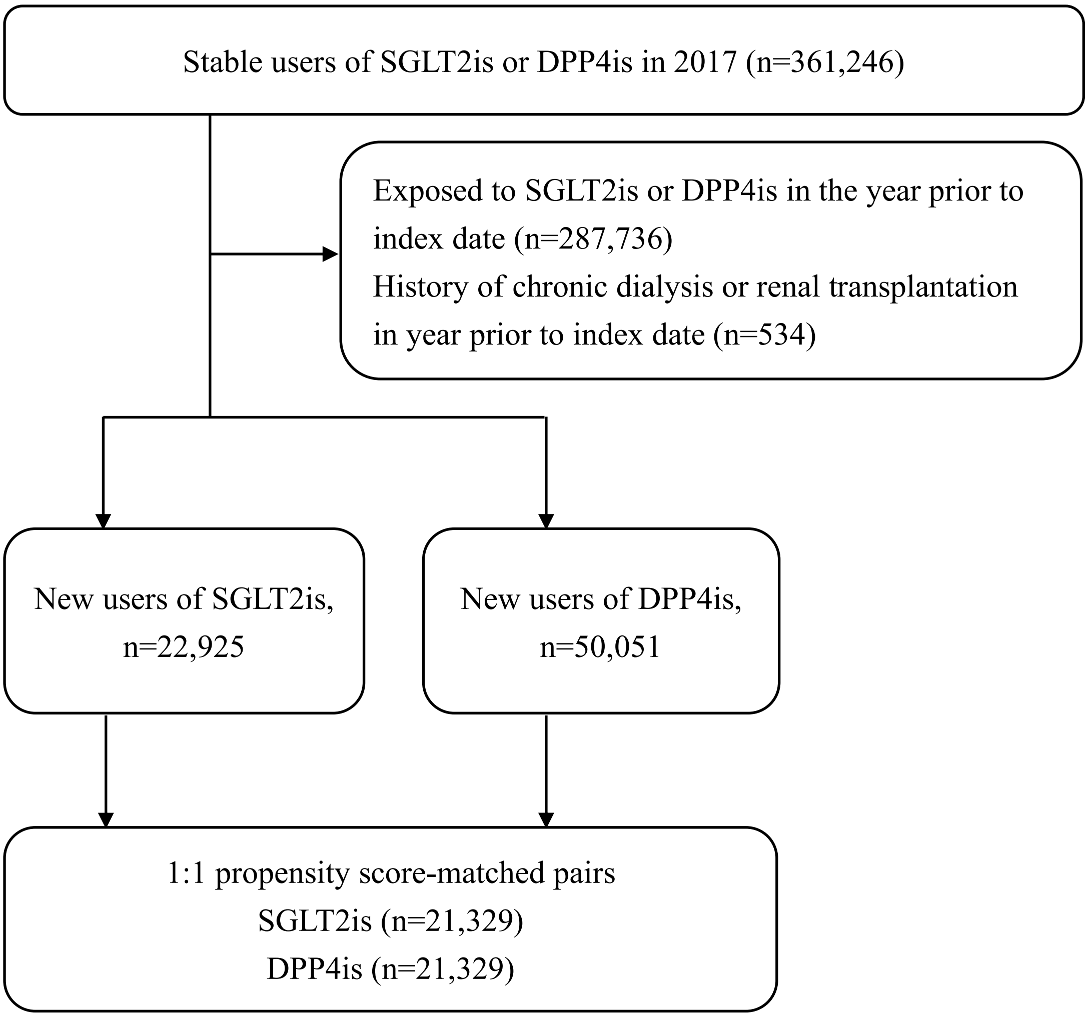

Abbreviations: SGLT2is, sodium glucose cotransporter 2 inhibitors; DPP4is, dipeptidyl peptidase 4 inhibitors.

Note: Index date refers to the initiation date of the study drug (SGLT2is or DPP4is) in 2017.

Supplementary Figure 2. Kernel density curves of propensity score distributions for two study groups (SGLT2is and DPP4is) before and after matching

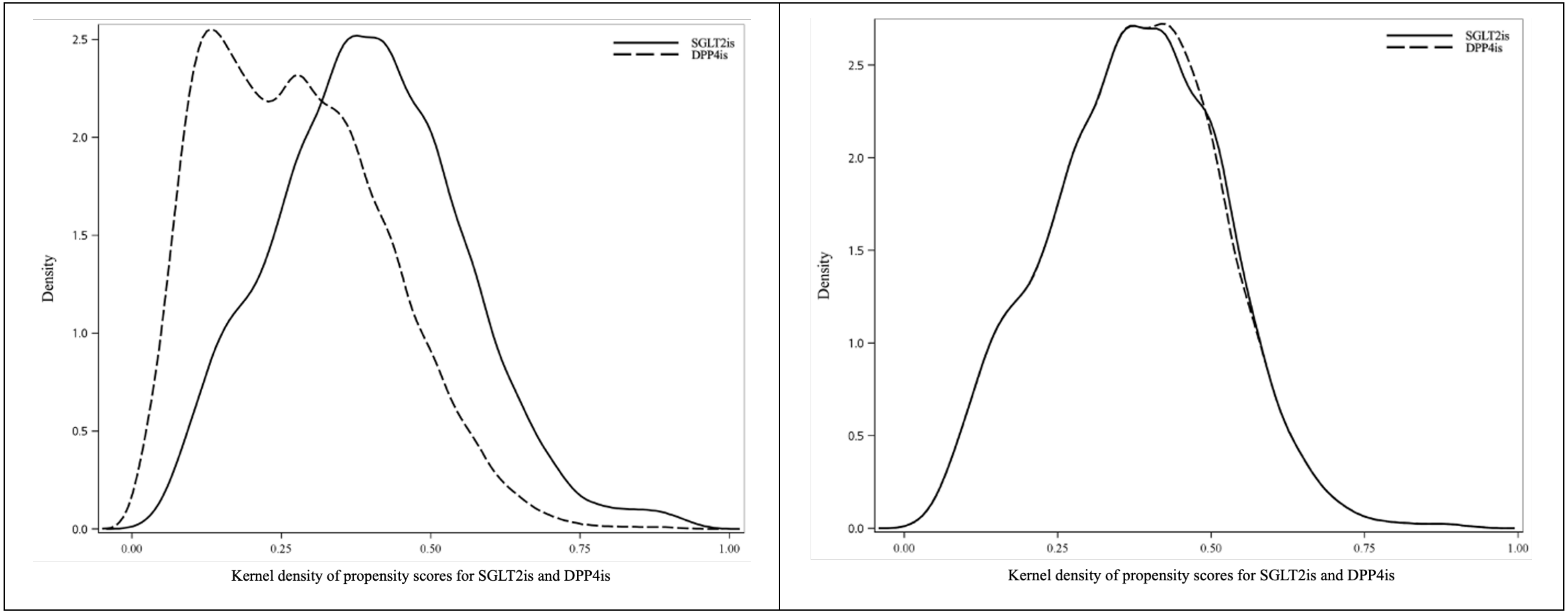

Note: Propensity score for each subject in study groups (i.e., SGLT2is and DPP4is) was estimated using logistic regression model analysis in which treatment status (i.e., SGLT2is versus DPP4is) was a dependent variable and the patient characteristics in the following six categories were independent variables:

(1) Demographics: age at the index date (measured at categorical levels, i.e., age < 45 years old, 45 ≤ age < 55 years old, 55 ≤ age < 65 years old, 65 ≤ age < 75 years old, age ≤ 75 years old), sex, duration of diabetes at the index date,

(2) Previous exposure to glucose-lowering agents (GLAs) in the year before the index date: the number of GLAs and types of GLAs (e.g., metformin, sulfonylureas, meglitinides, thiazolidinediones, acarbose, glucagon-like peptide-1 receptor agonists, insulins) subjects were exposed to within one year before the index date,

(3) Diabetes-related complications measured in the year before the index date: nephropathy, neuropathy, retinopathy, peripheral vascular disease, cerebrovascular diseases (i.e., transient ischemia attack, ischemic stroke, hemorrhagic stroke), cardiovascular diseases (i.e., heart failure, acute myocardial infarction, ischemic heart disease, arteriosclerotic cardiovascular disease metabolic, arrhythmia, cardiac arrest, cardiogenic shock), and metabolic complications (i.e., diabetic ketoacidosis, hypoglycemia),

(4) Comorbidities measured in the year before the index date: hypertension, hyperlipidemia, mental disorders (i.e., anxiety, bipolar disorders, post-traumatic stress disorder, schizophrenia, depression), and musculoskeletal disorders (i.e., osteoarthritis, low back pain),

(5) Surrogate indicators for renal function: participation in the pre-end-stage renal disease program within one year before the index date, medications with contraindications for patients with renal insufficiency (i.e., metformin, acarbose, sulfonylureas) prescribed within 90 days before the index date, and

(6) Exposure to cardiovascular disease-related medications within one year before the index date: lipid-lowering medications, alpha-blockers, beta-blockers, renin-angiotensin-aldosterone system agents, diuretics, calcium channel blockers, antiarrhythmics, cardiac glycosides, vasodilators, antiplatelets, and anticoagulants.

Supplementary Figure 3. Flowchart of study selection in a systematic review for clinical outcomes of SGLT2is versus DPP4is in Asian populations with type 2 diabetes

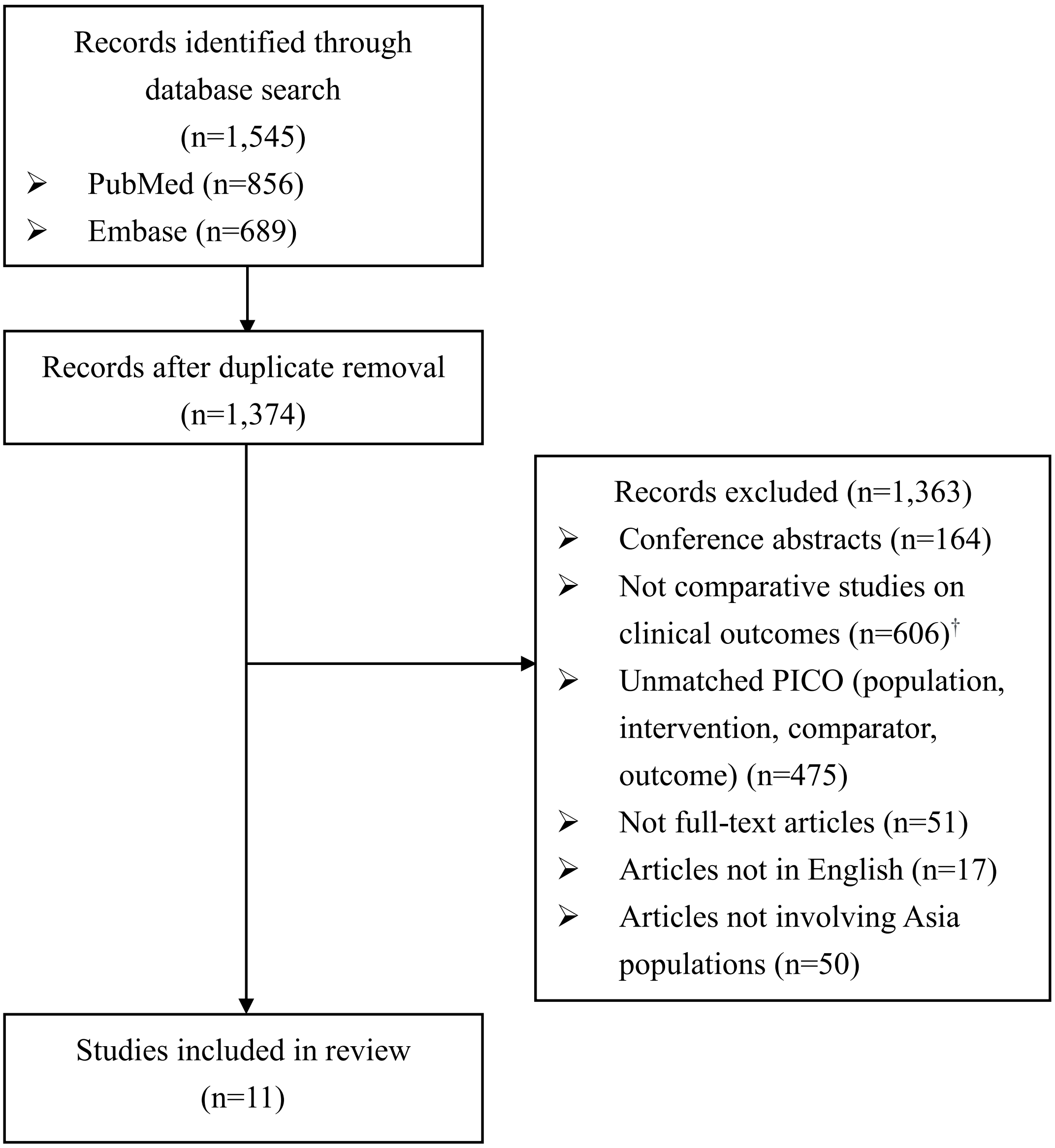

[†]Articles included case reports, reviews, commentaries, editorials, study protocols, guidelines, consensus studies, pharmacokinetic or pharmacodynamic studies, and economic evaluations (e.g., cost-effectiveness analysis, budget impact analysis).
